# Outcomes of extended versus standard lymphadenectomy in pancreatoduodenectomy for pancreatic cancer: systematic review and meta-analysis

**DOI:** 10.3389/fonc.2025.1622966

**Published:** 2025-06-27

**Authors:** Yu-Chun Xu, Yin-Hao Shi, Xiao-Feng Li

**Affiliations:** ^1^ Department of Gastroenterology, the Fifth Affiliated Hospital of Sun Yat-sen University, Zhuhai, China; ^2^ Department of Hepatobiliary Surgery and Liver Transplantation, the Fifth Affiliated Hospital of Sun Yat-sen University, Zhuhai, China

**Keywords:** pancreatic cancer, lymphadenectomy, prognosis, complications, meta-analysis

## Abstract

**Background:**

Pancreatic cancer has a poor prognosis, and surgical resection is the only curative option. Extended lymphadenectomy (EPD) during pancreatoduodenectomy may improve staging and reduce recurrence, but its survival benefits over standard lymphadenectomy (SPD) remain controversial.

**Methods:**

A systematic search of PubMed, Embase, Web of Science, and the Cochrane Library was conducted on March 25, 2025. All studies that met the inclusion criteria were subjected to quality assessment and subsequently analyzed by meta-analytical methods.

**Results:**

Nine RCTs involving 1382 patients were analyzed. No significant differences were observed between EPD and SPD in OS (HR = 1.09, *p* = 0.384), DFS (HR = 1.08, *p* = 0.506), or recurrence (78.05% vs. 79.64%, *p* = 0.295). EPD retrieved more positive lymph nodes (MD = 0.66, *p* = 0.008), but did not improve prognosis. Postoperative morbidity (38.49% vs. 33.27%, *p* = 0.072), mortality (1.97% vs. 1.33%, *p* = 0.589), transfusion volume (MD = -31.27, *p* = 0.469), and hospital stay (MD = -0.15, *p* = 0.917) were comparable, though EPD increased operative time (MD = 53.24, *p* < 0.001).

**Conclusions:**

EPD reduces lymph node recurrence without improving OS or DFS, suggesting limited prognostic benefit. Its application in pancreatic cancer should be carefully considered.

**Systematic review registration:**

https://www.crd.york.ac.uk/prospero, identifier CRD42024594566.

## Introduction

Pancreatic cancer remains one of the most lethal malignancies, with surgical resection being the only potentially curative treatment for localized disease (1, 2). However, the high risk of locoregional and distant recurrence even after curative-intent surgery raises concerns regarding the long-term therapeutic benefits of resection alone (3). Lymph nodes beyond the standard resection boundaries are potential sites for microscopic metastases, which may contribute to early relapse (4). Given that lymph node involvement is a key prognostic factor in pancreatic cancer, accurate assessment of nodal status is essential for staging and guiding postoperative therapeutic decisions (5). Pancreaticoduodenectomy (PD) remains the standard surgical procedure for tumors located in the pancreatic head. Two primary lymphadenectomy strategies are employed during PD: standard lymphadenectomy (SPD) and extended lymphadenectomy (EPD) (6). EPD involves a more comprehensive dissection of regional lymph nodes and peripancreatic nerve plexuses, aiming to enhance staging accuracy, reduce locoregional recurrence, and potentially improve oncologic outcomes by increasing the likelihood of removing occult metastases. However, EPD is technically more demanding, associated with prolonged operative time, and carries a higher risk of postoperative complications such as pancreatic fistula, delayed gastric emptying, and intra-abdominal abscess (7). Over the past decades, multiple randomized controlled trials (RCTs) have compared SPD and EPD in terms of survival benefit and surgical morbidity. Despite the theoretical advantages of EPD, current evidence remains inconclusive, and the optimal extent of lymphadenectomy during PD continues to be a subject of debate in the surgical management of pancreatic cancer.

Previous RCTs have yielded conflicting results regarding the survival benefit and complication rates associated with different extents of lymphadenectomy. Wang et al. reported superior 2-year overall survival (OS) in the SPD group, with a comparable incidence of postoperative complications between SPD and EPD groups (8). However, in a subsequent multicenter RCT, they found that while EPD improved the accuracy of TNM staging, it conferred no long-term survival advantage and was associated with reduced 1-year survival—primarily attributed to the lower completion rate of adjuvant therapy in the EPD cohort (9). Adding further complexity to the debate, a large-scale multicenter RCT conducted by Lin et al. (10) demonstrated that EPD significantly prolonged disease-free survival (DFS) without increasing the incidence of postoperative complications compared to SPD. Notably, the benefit of EPD was not observed across all patients with resectable pancreatic cancer. Subgroup analysis revealed that patients with preoperative serum CA19–9 levels < 200 U/mL experienced significantly improved OS and DFS following EPD. Based on these findings, the investigators recommended that patients with stage I–II pancreatic cancer and preoperative CA19–9 levels < 200 U/mL should undergo EPD followed by adjuvant chemotherapy, whereas those with CA19–9 levels ≥200 U/mL should receive SPD followed by postoperative chemotherapy. Nonetheless, despite multiple RCTs, no clear consensus has been established regarding the optimal extent of lymphadenectomy, and a comprehensive, up-to-date synthesis of the available evidence is still lacking.

To resolve these inconsistencies, the present meta-analysis systematically integrates data from recent high-quality RCTs to compare the clinical efficacy of EPD versus SPD in patients with pancreatic cancer. By comprehensively evaluating survival outcomes, recurrence patterns, and postoperative complication rates, this study seeks to elucidate the oncological value of EPD. Ultimately, the analysis aims to provide an updated and evidence-based assessment of surgical strategies, thereby informing clinical decision-making and improving prognostic outcomes in pancreatic cancer management. The findings of this study may help refine surgical guidelines and facilitate risk-adapted lymphadenectomy strategies in clinical practice.

## Materials and methods

### Literature research

Two authors independently conducted a comprehensive literature search in PubMed, Embase, Web of Science, and the Cochrane Library on March 25, 2025, without language restrictions. The search strategy combined both Medical Subject Headings (MeSH) and free-text terms, using the following key terms: “pancreatic neoplasms”, “pancreaticoduodenectomy”, and “lymph node excision”. Detailed search strategies were provided in the **Supplementary File**. This study was registered in the PROSPERO database (Registration Number: CRD42024594566).

### Inclusion and exclusion criteria

Inclusion criteria were as follows: (1) Studies involving patients with a confirmed pathological diagnosis of pancreatic cancer; (2) Availability of operative outcomes, including survival, recurrence, mortality, morbidity, number of positive lymph nodes resected, and detailed surgical information; (3) Study design limited to prospective randomized controlled trials.

The exclusion criteria were as follows: (1) Studies involving patients diagnosed with distal bile duct, ampullary, or duodenal cancers; (2) Duplicates, review articles, meta-analyses, case reports, single-arm studies, and conference abstracts; (3) Studies for which the full-text version could not be retrieved.

### Definition of lymphadenectomy and outcomes

The definitions of EPD and SPD as used in the literature were provided in the **Supplementary Table S1**. The primary endpoints of this meta-analysis were OS and DFS. OS was defined as the duration from the date of randomization to the date of death from any cause. DFS was defined as the time from randomization to either disease recurrence or death from any cause, whichever occurred first. The secondary outcomes included recurrence rates, number of harvested positive lymph nodes, postoperative morbidity (including pancreatic fistula, bile leakage, delayed gastric emptying, severe sepsis, intra-abdominal abscess, and postoperative hemorrhage), postoperative mortality, intraoperative transfusion volume, operative time, reoperation rate, and length of postoperative hospital stay.

### Data extraction

Data extraction was independently conducted by two authors (YC Xu and YH Shi). The literature screening process began with automated deduplication using EndNote 20. This was followed by an initial screening of titles and abstracts to exclude irrelevant or non-eligible studies. Articles that met the preliminary inclusion criteria were then subjected to full-text review based on predefined eligibility parameters. Data extraction followed a standardized protocol, with essential study characteristics systematically recorded in an electronic data matrix. Any discrepancies between reviewers were resolved through discussion to ensure consistency and accuracy. Extracted information included: first author, year of publication, country, study duration, study design, intervention groups, number of patients, patient demographics (age, sex), use of adjuvant therapy, surgical techniques, pathological characteristics, survival outcomes, recurrence, morbidity and mortality rates.

### Quality assessment

Methodological quality was assessed using the Cochrane Collaboration’s Risk of Bias Tool (11). The following domains were evaluated (1): bias arising from the randomization process (2); bias due to deviations from intended interventions (3); bias due to missing outcome data (4); bias in outcome measurement; and (5) bias in the selection of reported results.

### Statistical analysis

The data were processed according to the methods mentioned previously (12, 13). Dichotomous outcomes were analyzed using RR, and time-to-event or continuous outcomes using HR or MD, all with 95% CIs. Heterogeneity across studies was assessed using the Q-test, and the degree of heterogeneity was quantified by the I² statistic, with values of 25%, 50%, and 75% indicating low, moderate, and high heterogeneity, respectively (14). A fixed-effects model was applied when heterogeneity was low; otherwise, a random-effects model was used. All statistical analyses and plots generation were conducted using R software (version 4.4.1) with the “metafor” and “robvis” packages. Survival data were extracted using Engauge Digitizer (version 12.1). When survival data were available only in the form of Kaplan–Meier curves, they were estimated using the method described by Tierney et al (15). Publication bias was assessed using Egger’s regression test for funnel plot asymmetry, implemented via a mixed-effects or fixed-effects meta-regression model with standard error as the predictor. A *p* -value < 0.05 was considered statistically significant.

## Results

### Characteristics of included studies

**Figure 1:**
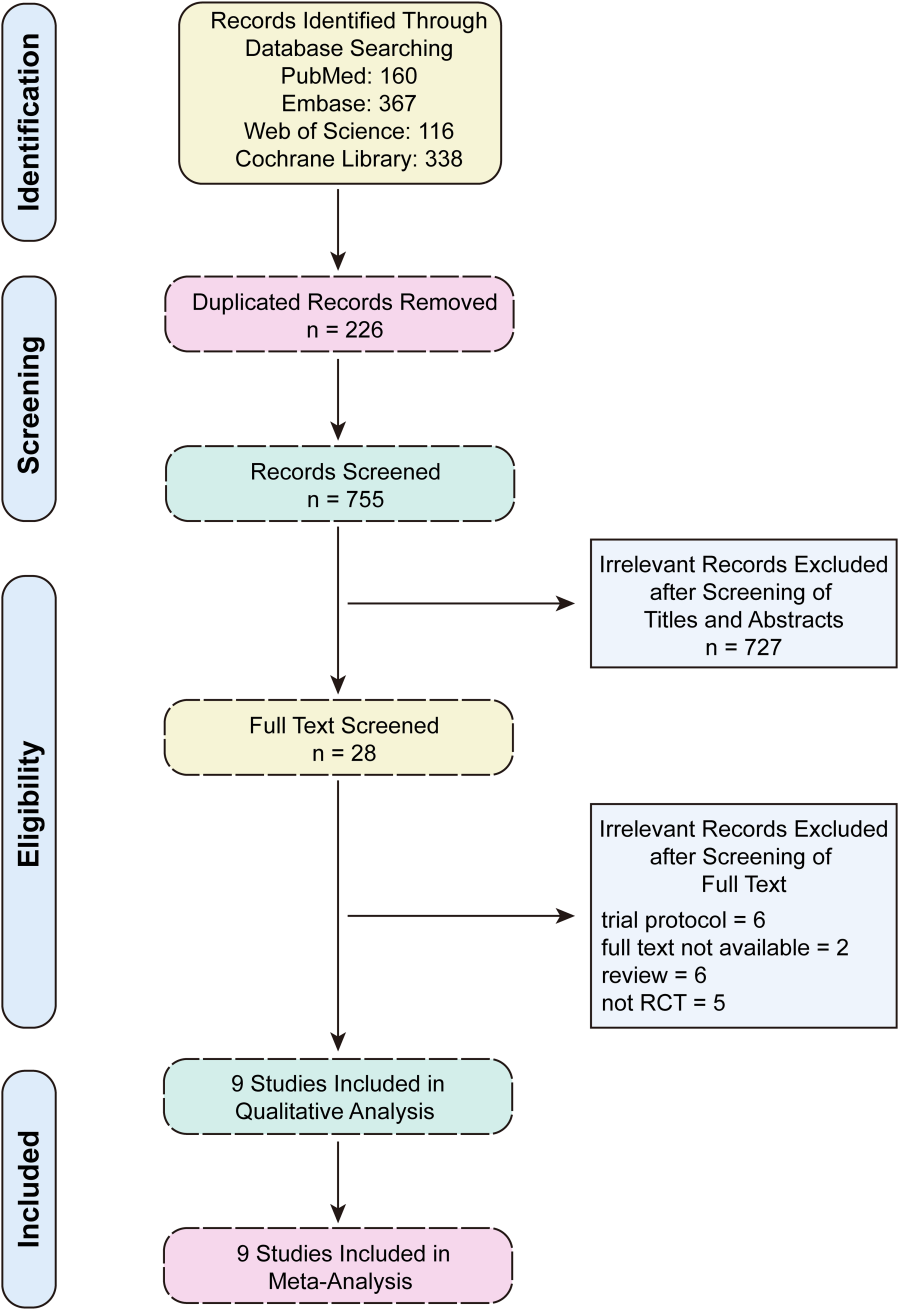
The flowchart of the literature screening process.

A total of 981 articles were initially identified through a comprehensive systematic search of PubMed, Embase, Web of Science, and the Cochrane Library. After removing duplicates and screening titles, abstracts, and full-texts, nine randomized controlled trials (RCTs) met the inclusion criteria (**Figure 1**). These studies included 696 patients in the EPD group and 686 patients in the SPD group. It was noteworthy that two publications originated from the same RCT, with one study reporting 2-year survival outcomes (16) and the other evaluating 5-year survival outcomes (17). The baseline characteristics of the nine included studies were summarized in **Table 1** and the tumor characteristics were presented in **Supplementary Table S2**. Based on methodological quality assessment, two studies (16, 17) were classified as high quality, while the remaining seven studies (8–10, 18–21) were rated as moderate quality (**Supplementary Figure S1**).

**Table 1:** Characteristics of patients of included studies.

Author	Year	Country	Span of study	Type of study	Study group	Number of patients	Age	Gender(M/F)	Adjuvant therapy
Pedrazzoli et al. (21)	1998	Italy	1991-1994	prospective	Standard	40	62 (26–81) *	27/13	0
					Extended	41	59.2 (31-77) *	25/16	0
Farnell et al. (18)	2005	USA	1997-2003	prospective	Standard	40	66 (32-84) *	20/20	30
					Extended	39	67 (38-80) *	21/18	26
Nimura et al. (20)	2012	Japan	2000-2003	prospective	Standard	51	62.7**	NA	0
					Extended	50	62.9**	NA	0
Jang et al. (16)	2014	Korea	2006-2009	prospective	Standard	83	62.0 ± 8.7***	49/34	NA
					Extended	86	63.4 ± 9.5***	44/42	NA
Ignjatovic et al. (19)	2017	Serbia	2007-2010	prospective	Standard	30	65.0 ± 4.9***	18/12	0
					Extended	30	59.5 ± 7.0***	14/16	0
Jang et al. (17)	2017	Korea	2006-2009	prospective	Standard	83	62.0 ± 8.7***	49/34	NA
					Extended	86	63.4 ± 9.5***	44/42	NA
Wang et al. (8)	2021	China	2016-2018	prospective	Standard	79	59.5 ± 10.6***	48/31	32
					Extended	74	57.2 ± 9.9***	45/29	29
Wang et al. (9)	2023	China	2016-2018	prospective	Standard	81	63.44 ± 9.27***	55/26	81
					Extended	89	60.78 ± 9.46***	55/34	89
Lin et al. (10)	2023	China	2012-2017	prospective	Standard	199	60 (28-79) *	111/88	173
					Extended	201	59 (19-79) *	117/84	170

NA, not available; *: median(range), **: mean; ***: mean ± SD,

### Primary outcomes

#### Overall survival

**Figure 2:**
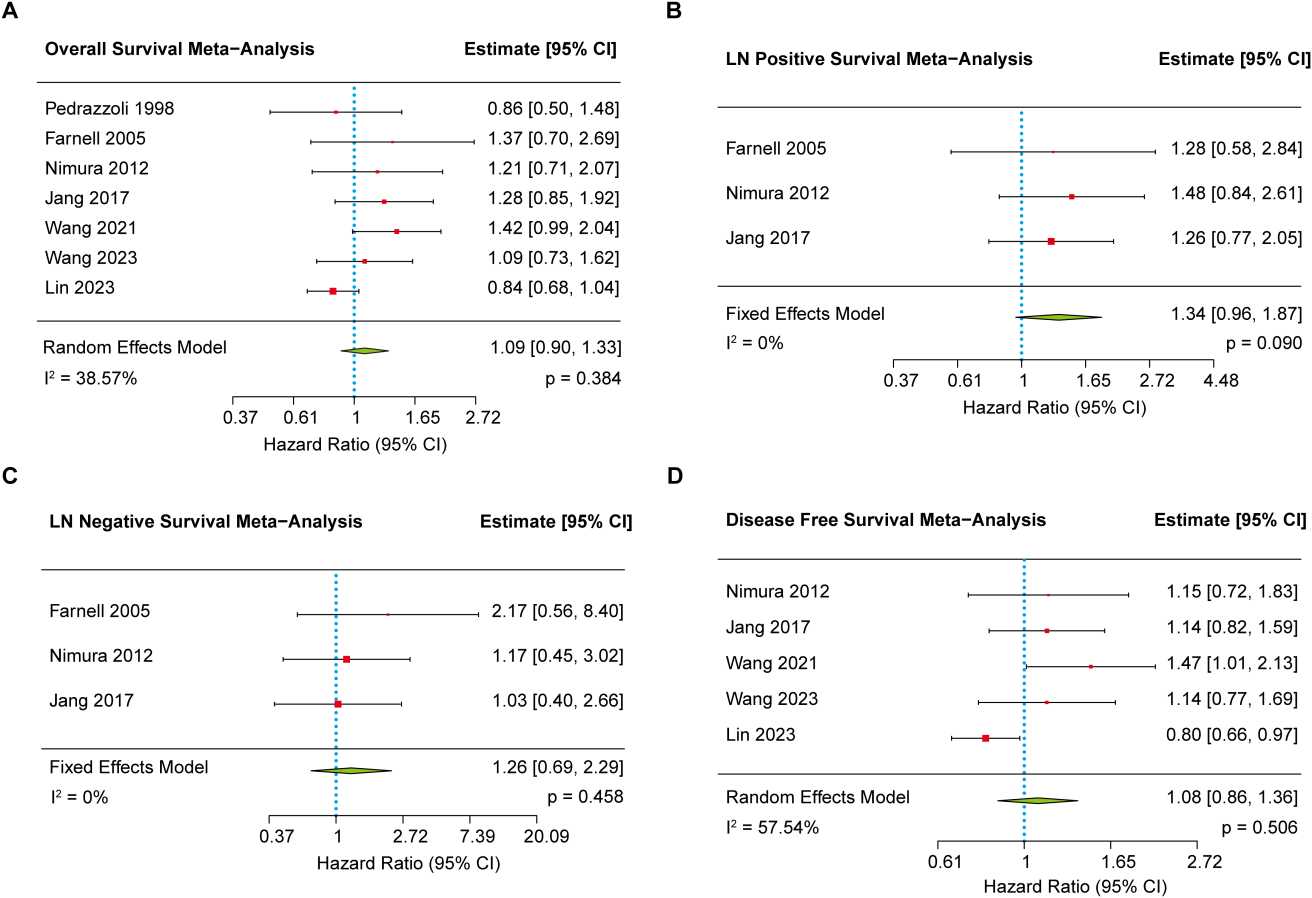
The forest plot showing the primary outcomes between EPD and SPD group. **(A)** The meta-analysis of overall survival. **(B)** The meta-analysis of overall survival in positive lymph node patients. **(C)** The meta-analysis of overall survival in negative lymph node patients. **(D)** The meta-analysis of disease-free survival.

Seven studies (8–10, 17, 18, 20, 21) reported data on OS and were included in this meta-analysis. Moderate heterogeneity was observed across studies (I^2^ = 38.57%); therefore, a random-effects model was applied. The pooled results demonstrated no statistically significant difference in OS between the EPD and SPD groups (HR = 1.09, 95% CI: 0.90–1.33, *p* = 0.384) (**Figure 2A**). Assessment of publication bias using Egger’s test revealed no statistically significant funnel plot asymmetry (*p* = 0.190).

Given the presence of heterogeneity, a sensitivity analysis was performed to identify potential sources. Upon exclusion of the study by Lin et al. (10), heterogeneity was markedly reduced, and the updated analysis revealed a statistically significant OS benefit in the SPD group compared to the EPD group (HR = 1.21, 95% CI: 1.01–1.46, *p* = 0.044) (**Supplementary Figure S2**). Moreover, given that geographic region and surgical approach are clinically relevant sources of heterogeneity, we further conducted meta-regression analyses to investigate their impact. The results indicated neither geographic region (*p* = 0.838) nor adherence to 2014 consensus guidelines (surgical approach) (*p* = 0.612) showed a statistically significant association with overall survival, indicating that these factors had minimal impact on survival outcomes across studies (**Supplementary Figure S3**).

To further explore whether lymph node status (positive or negative) influenced survival outcomes, a subgroup analysis was conducted using three studies (17, 18, 20) that reported survival stratified by lymph node status. Heterogeneity was low for both subgroups (I² = 0%). The pooled results indicated no significant survival difference between EPD and SPD, regardless of nodal involvement (**Figures 2B, C**). Egger’s tests for both subgroups also showed no evidence of publication bias (*p* = 0.981 for positive nodes; *p* = 0.377 for negative nodes).

#### Disease free survival

Five studies (8–10, 17, 20) reported DFS and were included in the meta-analysis. Moderate heterogeneity was observed (I² = 57.54%), and thus a random-effects model was applied. The pooled analysis demonstrated no statistically significant difference in DFS between the EPD and SPD groups (HR = 1.08, 95% CI: 0.86–1.36, *p* = 0.506) (**Figure 2D**). Egger’s regression test indicated significant funnel plot asymmetry (*p* = 0.005), suggesting potential publication bias. However, trim-and-fill analysis estimated no missing studies on the right side of the funnel plot (SE = 0.4071), indicating limitations in adjusting for the observed asymmetry despite evidence of bias. To explore the source of heterogeneity, a sensitivity analysis and meta-regression were performed. Upon exclusion of Lin et al.’s study (10), heterogeneity was substantially reduced, and the updated analysis revealed a statistically significant DFS benefit in favor of the SPD group (HR = 1.22, 95% CI: 1.01–1.48, *p* = 0.041) (**Supplementary Figure S4A–E**). Furthermore, since the 5 included studies were conducted in geographically similar regions, we used surgical technique—specifically the definition of EPD—as the sole moderator in the meta-regression analysis. The result showed no significant association between adherence to 2014 consensus guidelines (definition of EPD) and hazard ratios (*p* = 0.769) (**Supplementary Figure S4F**).

### Secondary outcomes

#### Recurrence

**Figure 3:**
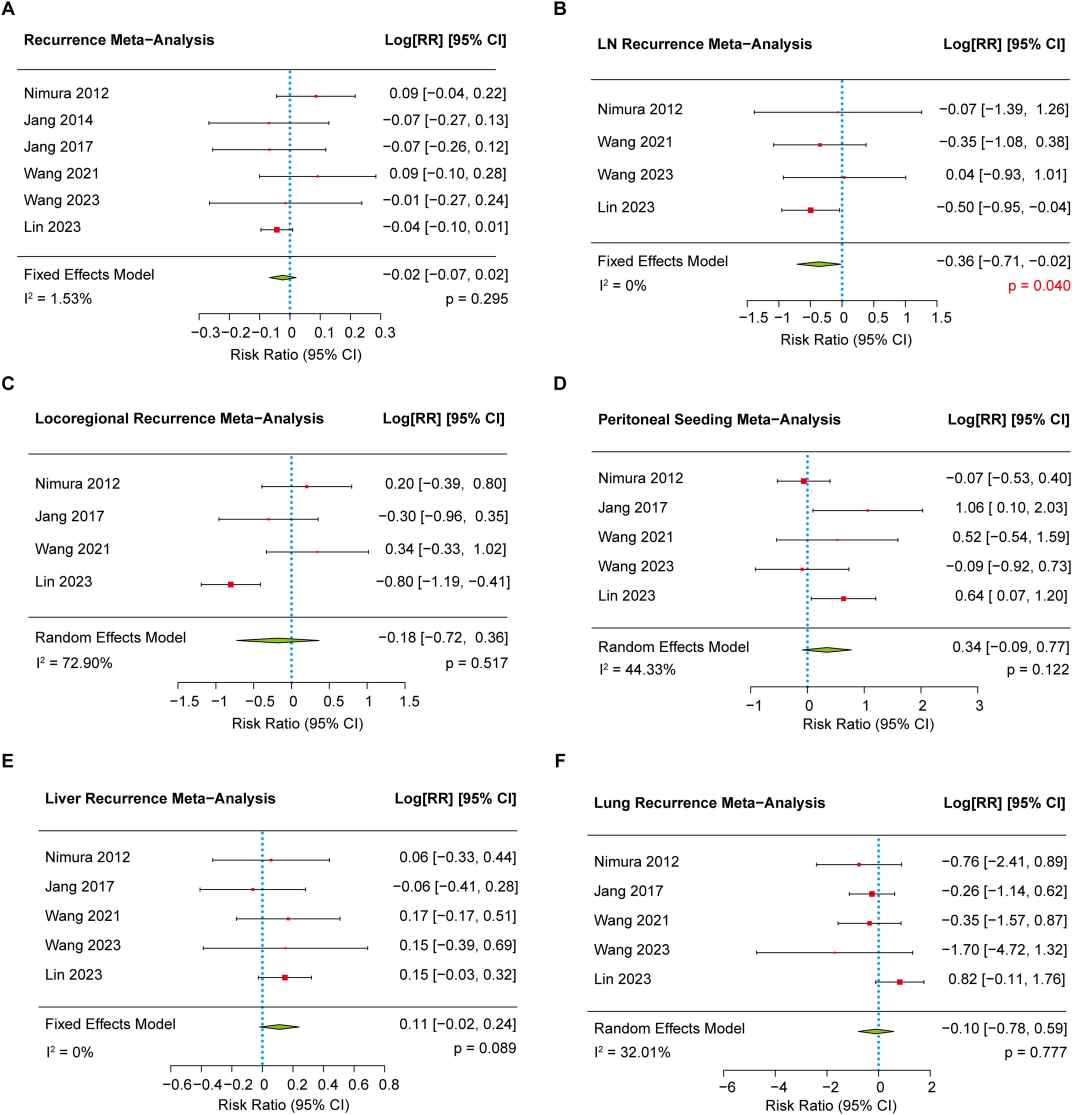
The forest plot showing the recurrent rate between EPD and SPD group. **(A)** The meta-analysis of recurrent rate. **(B)** The meta-analysis of lymph node recurrent rate. **(C)** The meta-analysis of locoregional recurrent rate. **(D)** The meta-analysis of peritoneal seeding rate. **(E)** The meta-analysis of liver recurrent rate. **(F)** The meta-analysis of lung recurrent rate.

Six studies (8–10, 16, 17, 20) reported data on recurrence and were included in the meta-analysis. The pooled analysis showed no significant difference in the overall recurrence rate between the EPD and SPD groups (78.05% vs. 79.64%, *p* = 0.295) (**Figure 3A**). Further subgroup analyses were conducted to evaluate specific patterns of recurrence, including lymph node recurrence, locoregional recurrence, peritoneal seeding, liver metastasis, and lung metastasis. Among these, the lymph node recurrence rate (8–10, 20) was found to be significantly lower in the EPD group compared to the SPD group (11.78% vs. 17.05%, *p* = 0.040) (**Figure 3B**). However, no significant differences were observed between groups for other recurrence patterns (**Figures 3C–F**). No evidence of publication bias (*p* = 0.373) was found.

#### Harvested positive lymph nodes

Three studies (9, 10, 16) reported data on the number of harvested positive lymph nodes and were included in the meta-analysis. The pooled analysis showed that the EPD group had significantly more positive lymph nodes harvested compared to the SPD group with low heterogeneity (MD = 0.66, 95% CI: 0.17 to 1.15, *p* = 0.008) (**Supplementary Figure S5**). No publication bias was detected by Egger’s test (*p* = 0.765).

#### Postoperative mortality

**Figure 4:**
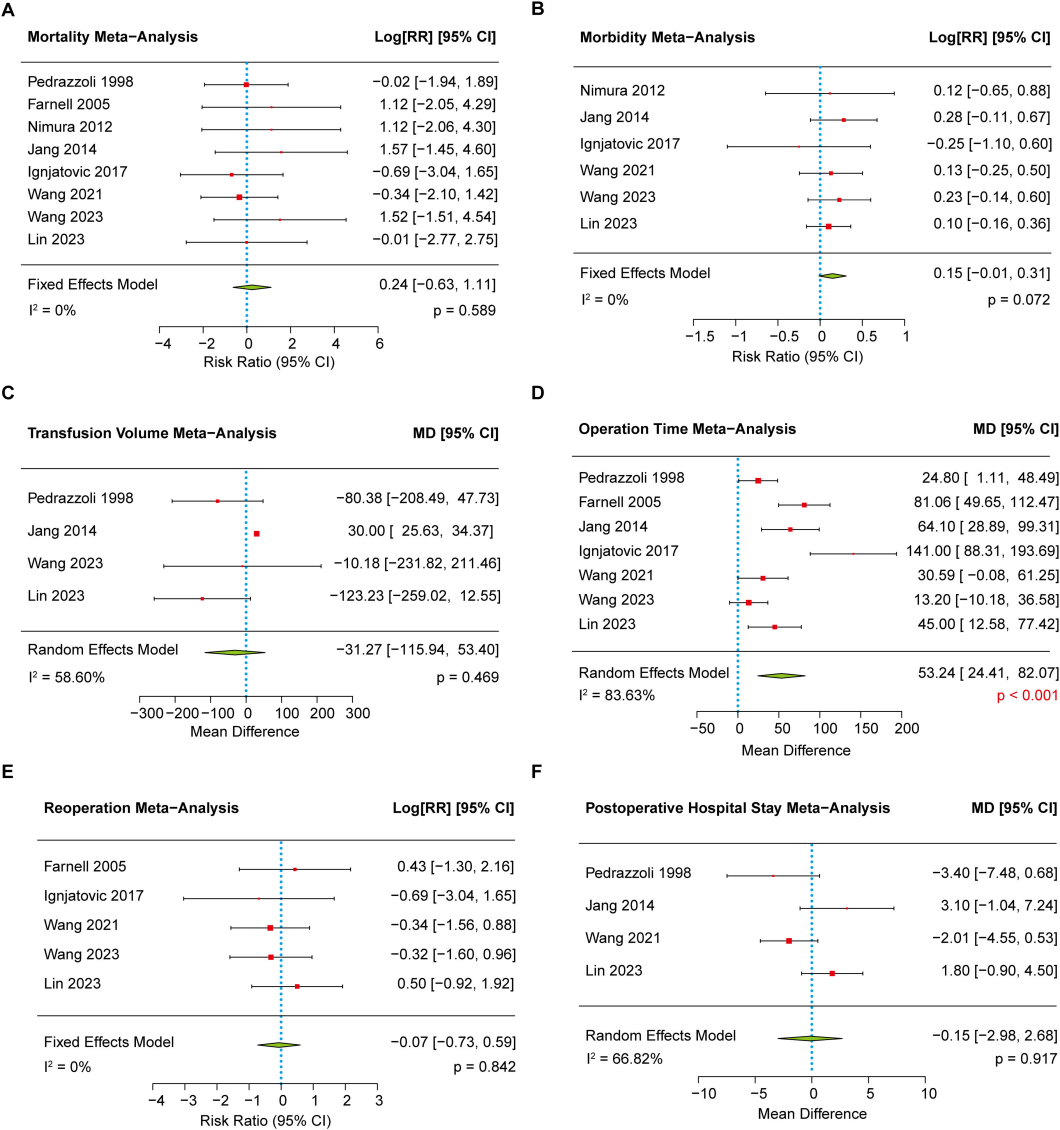
The forest plot showing the other secondary outcomes between EPD and SPD group. **(A)** The meta-analysis of postoperative mortality. **(B)** The meta-analysis of postoperative morbidity. **(C)** The meta-analysis of transfusion volume. **(D)** The meta-analysis of operation time. **(E)** The meta-analysis of reoperation rate. **(F)** The meta-analysis of length of postoperative hospital stays.

Eight studies (8–10, 16, 18–21) reported data on postoperative mortality and were included in the meta-analysis. Heterogeneity was negligible (I² = 0), and a fixed-effects model was applied accordingly. No significant difference was observed between the EPD and SPD groups (1.97% vs. 1.33%, *p* = 0.589) (**Figure 4A**). Egger’s test indicated no significant publication bias (*p* = 0.159).

#### Postoperative morbidity

Six clinical trials (8–10, 16, 19, 20) reported data on postoperative morbidity between the EPD and SPD groups, with low heterogeneity (I² = 0). Although the incidence of postoperative morbidity was slightly higher in the EPD group (38.49% vs 33.27%), the difference was not statistically significant (*p* = 0.072) (**Figure 4B**). Further analysis of specific complications—including pancreatic fistula, bile leakage, delayed gastric emptying, severe sepsis, intra-abdominal abscess, and postoperative hemorrhage—also revealed no significant differences between the two groups (**Table 2**). No significant funnel plot asymmetry (*p* = 0.663) was found.

**Table 2:** The meta-analysis results of postoperative complications.

Complications	Study	Heterogeneity	Model	Log[RR]	95% CI	p value
Pancreatic fistula	7	0	fixed-effects	0.04	(-0.31, 0.39)	0.806
Bile leakage	3	46.22%	random-effects	0.53	(-0.61, 1.67)	0.363
Delayed gastric empty	6	0	fixed-effects	0.24	(-0.14, 0.62)	0.212
Severe sepsis	3	0	fixed-effects	0.27	(-0.78, 1.31)	0.618
Intra-abdominal abscess	5	0	fixed-effects	0.09	(-0.31, 0.49)	0.654
Postoperative hemorrhage	6	0	fixed-effects	-0.04	(-0.56, 0.49)	0.895

#### Transfusion volume

Four studies (9, 10, 16, 21) reported transfusion volume, showing no overall difference between EPD and SPD [MD, 95% CI: –31.27 (–115.94 to 53.40), *p* = 0.469)]despite moderate heterogeneity (I² = 53.60%) (**Figure 4C**). Sensitivity analysis indicated EPD had lower transfusion volume [MD, 95% CI: –86.99 (–172.89 to –1.09), *p* = 0.047] (**Supplementary Figure S6A**) after excluding one outlier study (16). However, sensitivity analyses excluding other studies resulted in increased heterogeneity, and the meta-analyses continued to show no significant differences between the groups (**Supplementary Figures S6B**–**S6D**). In addition, funnel plot asymmetry and trim-and-fill analysis suggested possible publication bias and overestimation of effects (*p* = 0.014).

#### Operation time

Seven studies (8–10, 16, 18, 19, 21) reported operative time, and a random-effects model showed that EPD significantly increased operation time compared to SPD [MD, 95% CI: 53.24 (24.41 to 82.07), *p* < 0.001] (**Figure 4D**) with high heterogeneity (I² = 83.63%). Egger’s test revealed substantial funnel plot asymmetry (p < 0.001), but trim-and-fill analysis suggested no missing studies, implying heterogeneity may stem from clinical or methodological differences rather than publication bias.

#### Reoperation

Five studies (8–10, 18, 19) reported reoperation rates with low heterogeneity (I² = 0). A fixed-effects model showed no significant difference between the EPD and SPD groups (3.93% vs. 4.20%, *p* = 0.842) (**Figure 4E**). Furthermore, Egger’s test showed no evidence of publication bias (*p* = 0.990), suggesting reliable results.

#### Postoperative hospital stays

Four studies (8, 10, 16, 21) were included to assess hospital stay, showing moderate heterogeneity (I² = 66.82%). A random-effects model was applied and showed no significant difference between EPD and SPD groups [MD 95% CI: –0.15 (–2.98 to 2.68), *p* = 0.917] (**Figure 4F**). In addition, Egger’s test using a mixed-effects model revealed no significant funnel plot asymmetry (*p* = 0.933).

## Discussion

Pancreaticoduodenectomy is widely recognized as the standard surgical procedure for pancreatic head cancer. In Japan, EPD has been proposed as a strategy to improve survival by removing lymph nodes that may harbor metastases beyond the standard dissection scope of SPD (4). In addition to the resection of lymph node stations 5, 6, 8a, 12b, 12c, 13, 14a, 14b, and 17, the EPD involves the resection of stations 9, 12p, 14c, 14d, 16a2, and 16b1, as outlined in the 2014 ISGPS consensus statement (22). Moreover, EPD requires the complete dissection and skeletonization of all soft tissues within the hepatoduodenal ligament, as well as resection of the right-sided celiac plexus and the nerve plexus around the superior mesenteric artery (SMA) (23).

Despite decades of accumulated clinical experience, a consensus on the optimal surgical strategy for lymph node management in pancreatic head cancer remains elusive, primarily due to the absence of a demonstrated OS benefit in previous trials comparing EPD with SPD. Apart from clinical trials, several meta-analyses also shown that EPD did not significantly improve OS, with postoperative morbidity rates comparable to those of conventional resection (24–26). However, these analyses often included older studies or trials with limited sample sizes, potentially affecting the robustness and generalizability of their conclusions. Notably, a recent large-scale RCT by Lin et al., which enrolled 400 patients with pancreatic cancer, provided new insights into the outcomes of SPD versus EPD (10). While consistent with earlier findings in showing no significant OS benefit with EPD, this trial uniquely reported a significantly prolonged DFS in the EPD group. Nevertheless, despite incorporating this high-quality RCT, our current meta-analysis did not confirm a statistically significant DFS advantage for EPD.

Emerging evidence indicates that pancreatic cancer often undergoes systemic dissemination prior to clinical detection, with occult micro-metastases present in most cases that appear localized on imaging (3, 27). This biological behavior renders anatomical staging alone insufficient for evaluating curative potential. The high incidence of lymph node metastasis significantly contributes to early postoperative recurrence and poor prognosis following pancreaticoduodenectomy for pancreatic cancer. As a result, more aggressive surgical strategies—including extended lymphadenectomy, en bloc resection of adjacent organs, and vascular resection—have been proposed to improve long-term outcomes. However, the survival benefit of extended lymphadenectomy remains controversial and continues to be a subject of active debate.

In our study, although the number of positive lymph nodes retrieved was significantly higher in the EPD group than in the SPD group [0.66 (0.17, 1.15), p = 0.008], and the rate of postoperative lymph node recurrence was significantly lower in the EPD group [11.78% vs. 17.05%, p = 0.040], these advantages in nodal clearance did not translate into improved survival outcomes [1.09 (0.90, 1.33), p = 0.384]. Notably, no significant survival benefit was observed in EPD patients with positive lymph nodes compared with their SPD counterparts [1.34(0.96, 1.87), *p* = 0.090]. These findings suggest that metastatic to certain lymph node stations may reflect systemic disease rather than a surgically curable focus, supporting the concept of ‘biological predeterminism’ in cancer progression (28). Therefore, adjuvant therapy, rather than extended resection, may be a more effective approach to improve long-term survival. The survival benefits of adjuvant chemotherapy following pancreatic resection for pancreatic cancer are well established, and it is recommended as standard care in most national guidelines. However, adjuvant therapy may confound the isolated impact of surgical intervention itself (29). Some researchers have proposed that postoperative survival in pancreatic cancer is largely determined by the efficacy of adjuvant chemotherapy rather than surgical technique alone (16, 17). Moreover, evidence suggests that patients with delayed postoperative lymphocyte recovery are more susceptible to recurrence and have worse prognoses (30). Interestingly, EPD has been associated with higher local recurrence rates, possibly due to heightened immunosuppression resulting from more extensive surgical trauma (20). These findings imply that the immunological effects of surgical stress may play a critical role in recurrence risk and long-term outcomes.

With regard to chemotherapy, Wang et al. (8) demonstrated that adjuvant chemotherapy significantly improved survival outcomes irrespective of the lymphadenectomy extent. Notably, patients in the SPD group had higher 2-year OS following chemotherapy, potentially due to better immune recovery. Completion of the full six-cycle chemotherapy regimen has been identified as an independent prognostic factor after surgery, and delayed initiation does not appear to compromise outcomes (9, 31–33). Similarly, Lin et al. (10) found that EPD patients who received adjuvant chemotherapy experienced significantly prolonged DFS, with no delay in treatment initiation. Moreover, median OS remained consistent with prior reports, while median DFS was modestly extended (29, 34–37). These findings support a more selective lymphadenectomy approach—targeting only histologically suspicious nodes—to reduce surgical trauma, preserve immune function, and maximize the effectiveness of adjuvant chemotherapy. Additionally, our study found that EPD retrieved more positive lymph nodes, potentially improving the accuracy of TNM staging (38). However, under-staging in the SPD group did not significantly affect prognosis, reinforcing that complete adjuvant chemotherapy may be more critical for long-term survival than extensive nodal dissection.

Notably, the recently published RCT by Lin et al. (10) demonstrated a significant DFS advantage for the EPD group compared to the SPD group, diverging from the findings of earlier studies. Specifically, EPD was associated with improved OS and DFS in patients with pancreatic head cancer who had a lower risk of systemic metastasis, as indicated by preoperative CA19–9 levels below 200U/mL. In patients with well to moderately differentiated tumors, EPD yielded a 6.2-month longer median OS and a 5.8-month longer median DFS compared to SPD. These findings further support that, in early-stage pancreatic cancer with low metastatic potential, EPD combined with retroperitoneal nerve dissection offers superior oncologic outcomes.

However, concerns regarding increased postoperative complications, such as diarrhea, have been raised. A previous study reported a higher incidence of diarrhea at three months following EPD (25). Interestingly, both Lin et al. (10) and Jang et al. (16) found no significant difference in diarrhea rates between the EPD and SPD groups. This discrepancy may be attributed to differences in surgical techniques. Earlier studies employed more aggressive dissection, involving complete clearance around the celiac axis and SMA. In contrast, Lin et al. used a modified approach involving limited dissection of nerves and soft tissue within a 270° arc on the right side of the celiac axis and SMA, potentially reducing gastrointestinal complications. To evaluate the robustness of Lin’s findings, we conducted a sensitivity analysis excluding their study, which revealed that SPD appeared more beneficial for DFS. Heterogeneity in meta-analyses typically arises from clinical, methodological, and statistical sources. Due to inherent limitations of meta-analysis, we were unable to access individual patient data from the included studies. Therefore, we considered geographic region and surgical technique as potential sources of heterogeneity and conducted meta-regression analyses using these two variables as moderators. Neither the surgical technique (definition of EPD based on consensus criteria) nor geographic region significantly influenced OS or DFS outcomes in our pooled analysis. Additionally, variation in statistical methods across studies may have contributed to the observed heterogeneity. Ultimately, we concluded that the main sources of heterogeneity related to OS and DFS did not significantly compromise the robustness of our findings.

Although the included studies were generally of high methodological quality, several limitations should be acknowledged: (1) Heterogeneity in surgical techniques, perioperative management, and definitions of EPD across studies may have introduced bias. (2) Variations in adjuvant therapy regimens and follow-up durations across studies could have influenced survival outcomes.(3) Some included RCTs had relatively small sample sizes, potentially limiting statistical power. (4) Despite comprehensive literature searches and funnel plot analyses, the possibility of publication bias cannot be entirely excluded.

In conclusion, although EPD may reduce postoperative lymph node recurrence and exhibits a safety profile comparable to SPD, current evidence does not support a clear survival benefit in terms of OS or DFS. Therefore, the application of EPD should be individualized and approached with caution. Based on our systematic review of recent RCTs and meta-analysis, we propose the following practical recommendations for surgical decision-making: (1) SPD should remain the standard approach, whereas EPD should not be applied indiscriminately; (2) preoperative imaging should be thoroughly evaluated for regional lymphadenopathy, with intraoperatively suspicious nodes selectively resected; (3) patients with preoperative CA19-9 < 200 U/mL may be considered for EPD, guided by intraoperative findings and clinical judgment; and (4) implementation of ERAS protocols is essential to promote recovery and ensure timely initiation and completion of adjuvant chemotherapy.

## Data Availability

The original contributions presented in the study are included in the article/**Supplementary Material**. Further inquiries can be directed to the corresponding author.
