# Supplementary material for: Outcomes of extended versus standard lymphadenectomy in pancreatoduodenectomy for pancreatic cancer: systematic review and meta-analysis
Source: Front Oncol. 2025 Jun 27;15:1622966. doi: 10.3389/fonc.2025.1622966 (PMC12245783; doi:10.3389/fonc.2025.1622966)
Supplement: Supplementary file 1 [file DataSheet1.docx]

Supplementary information for

**Outcomes of Extended versus Standard lymphadenectomy in Pancreatoduodenectomy for Pancreatic Cancer: Systematic Review and Meta-analysis**

**Authors:**

Yu-Chun Xu^1†^, Yin-Hao Shi^2†^, Xiao-Feng Li^1*^

^†^These authors contributed equally to this study.

**Affiliations:**

^1^Department of Gastroenterology, the Fifth Affiliated Hospital of Sun Yat-sen University, Zhuhai 519000, China

^2^Department of Hepatobiliary Surgery and Liver Transplantation, the Fifth Affiliated Hospital of Sun Yat-sen University, Zhuhai 519000, China

***Corresponding author:**

Xiao-Feng Li

Address: Department of Gastroenterology, the Fifth Affiliated Hospital of Sun Yat-sen University, Zhuhai 519000, China

Telephone: 86-0756-2528841

Email: zdwylxf@163.com

**Supplementary Materials – Index**

| **Supplementary Methods** |  |
| --- | --- |
| Retrieval Strategy | *pag. 3* |
| **Supplementary Figures** |  |
| Figure S1 | *pag. 6* |
| Figure S2 | *pag. 7* |
| Figure S3  Figure S4 | *pag. 9*  *pag. 10* |
| Figure S5 | *pag. 11* |
| Figure S6 | *pag. 12* |
| **Supplementary Tables** |  |
| Table S1 | *pag. 13* |
| Table S2 | *pag. 15* |

**Supplementary Methods**

**Retrieval Strategy**

1. **PubMed**

((Pancreaticoduodenectomy[mh] OR Pancreatectomy[mh] OR (Pancreatoduodenectom*[tiab] OR duodenopancreatectomy[tiab] OR pancreatectomy[tiab] OR hemipancreatectomy[tiab] OR hemi‐ pancreatectomy[tiab]) OR (pancrea*[tiab] AND (duodenectomy[tiab] OR resection[tiab] OR dissection[tiab] OR excision[tiab])) OR ((brunschwig[tiab] OR whipple[tiab]) AND (operation[tiab] OR resection[tiab]))) AND (Lymph Node Excision[mh] OR lymphadenectomy[tiab] OR lymphoadenectomy[tiab] OR ((lymph[tiab] OR node[tiab]) AND (dissect*[tiab] OR excis*[tiab] OR extirpate*[tiab] OR resect*[tiab] OR metastas*[tiab] OR retriev*[tiab]))) AND (clinicaltrial[Filter])) AND ((Pancreatic Neoplasms[mh] OR ((pancreatic[tiab] OR pancreas[tiab]) AND (adenocarcinoma[tiab] OR carcinoma[tiab] OR cancer[tiab] OR neoplasm*[tiab] OR tumor[tiab]))) AND (clinicaltrial[Filter])) Filters: Clinical Trial

1. **Embase**

**#1** ('pancreas cancer'/exp OR ((pancreatic OR pancreas) NEAR/3 (adenocarcinoma OR carcinoma OR cancer OR neoplasm* OR tumor)):ab,ti) NOT 'conference abstract'/it NOT 'case report'/de

**#2** ('pancreaticoduodenectomy'/exp OR 'pancreas resection'/exp OR (Pancreatoduodenectom* OR duodenopancreatectomy OR pancreatectomy OR hemipancreatectomy OR hemi‐ pancreatectomy):ab,ti OR (pancrea* NEAR/3 (duodenectomy OR resection OR dissection OR excision)):ab,ti OR ((brunschwig OR whipple) NEAR/3 (operation OR resection)):ab,ti) AND ('lymph node dissection'/exp OR lymphadenectomy:ab,ti OR lymphoadenectomy:ab,ti OR ('lymph node' NEAR/3 (dissect* OR excis* OR extirpate* OR resect* OR metastas* OR retriev*)):ab,ti) NOT 'conference abstract'/it NOT 'case report'/de

**#3** 'clinical trial'/de OR 'randomized controlled trial'/de OR 'controlled clinical trial'/de OR 'cohort analysis'/de OR 'prospective study'/de

**#1 and #2 and #3**

1. **Web of Science**

**#1** ((TS=("Pancreatic Neoplasms" OR "pancreatic tumor*" OR "pancreatic cancer"))OR ((TI=(pancreatic OR pancreas) OR AB=(pancreatic OR pancreas)) AND (TI=(adenocarcinoma OR carcinoma OR cancer OR neoplasm* OR tumo?r*) OR AB=(adenocarcinoma OR carcinoma OR cancer OR neoplasm* OR tumo?r*)))) AND DT=("Clinical Trial")

**#2** (TS=("Pancreaticoduodenectomy" OR "Pancreatectomy" OR "Whipple procedure" OR "Brunschwig operation") OR ((TI=("Pancreatoduodenectom*" OR "duodenopancreatectomy" OR "pancreatectomy" OR "hemipancreatectomy") OR AB=("Pancreatoduodenectom*" OR "duodenopancreatectomy" OR "pancreatectomy" OR "hemipancreatectomy")) AND (TI=("duodenectomy" OR "resection" OR "dissection" OR "excision") OR AB=("duodenectomy" OR "resection" OR "dissection" OR "excision")))) AND (TS=("Lymph Node Excision" OR "lymphadenectomy" OR "lymph node dissection") OR ((TI=("lymph" OR "node") OR AB=("lymph" OR "node")) AND (TI=("dissect*" OR "excis*" OR "resect*" OR "metastas*") OR AB=("dissect*" OR "excis*" OR "resect*" OR "metastas*")))) AND DT=("Clinical Trial")

**#1 and #2**

1. **Cochrane library**

**#1** MeSH descriptor: [Pancreatic Neoplasms] explode all trees

**#2** (pancreas):ti,ab,kw (Word variations have been searched)

**#3** (cancer):ti,ab,kw (Word variations have been searched)

**#4** (neoplas*):ti,ab,kw (Word variations have been searched)

**#5** (carcinoma*):ti,ab,kw (Word variations have been searched)

**#6** #2 AND (#3 OR #4 OR #5)

**#7** #1 OR #6

**#8** MeSH descriptor: [Pancreaticoduodenectomy] explode all trees

**#9** MeSH descriptor: [Pancreatectomy] explode all trees

**#10** (Pancreatoduodenectom*):ti,ab,kw (Word variations have been searched)

**#11** (duodenopancreatectomy):ti,ab,kw (Word variations have been searched)

**#12** (pancreatectomy):ti,ab,kw (Word variations have been searched)

**#13** (hemipancreatectomy):ti,ab,kw (Word variations have been searched)

**#14** (hemi-pancreatectomy):ti,ab,kw (Word variations have been searched)

**#15** (pancrea*):ti,ab,kw (Word variations have been searched)

**#16** (duodenectomy):ti,ab,kw (Word variations have been searched)

**#17** (resection):ti,ab,kw (Word variations have been searched)

**#18** (dissection):ti,ab,kw (Word variations have been searched)

**#19** (excision):ti,ab,kw (Word variations have been searched)

**#20** (brunschwig):ti,ab,kw (Word variations have been searched)

**#21** (whipple):ti,ab,kw (Word variations have been searched)

**#22** (operation):ti,ab,kw (Word variations have been searched)

**#23** (resection):ti,ab,kw (Word variations have been searched)

**#24** #8 OR #9 OR #10 OR #11 OR #12 OR #13 OR #14

**#25** #15 AND (#16 OR #17 OR #18 OR #19)

**#26** (#20 OR #21) OR (#22 OR #23)

**#27** #24 OR #25 OR #26

**#28** MeSH descriptor: [Lymph Node Excision] explode all trees

**#29** (lymphadenectomy):ti,ab,kw (Word variations have been searched)

**#30** (lymphoadenectomy):ti,ab,kw (Word variations have been searched)

**#31** (lymph):ti,ab,kw (Word variations have been searched)

**#32** (node):ti,ab,kw (Word variations have been searched)

**#33** (dissect*):ti,ab,kw (Word variations have been searched)

**#34** (excis*):ti,ab,kw (Word variations have been searched)

**#35** (extirpate*):ti,ab,kw (Word variations have been searched)

**#36** (resect*):ti,ab,kw (Word variations have been searched)

**#37** (metastas*):ti,ab,kw (Word variations have been searched)

**#38** (retriev*):ti,ab,kw (Word variations have been searched)

**#39** #28 OR #29 OR #30 OR ((#31 OR #32) AND (#33 OR #34 OR #35 OR #36 OR #37 OR #38))

**#40** #27 AND #39

**#41** #7 AND #40

**Supplementary Figures**

**
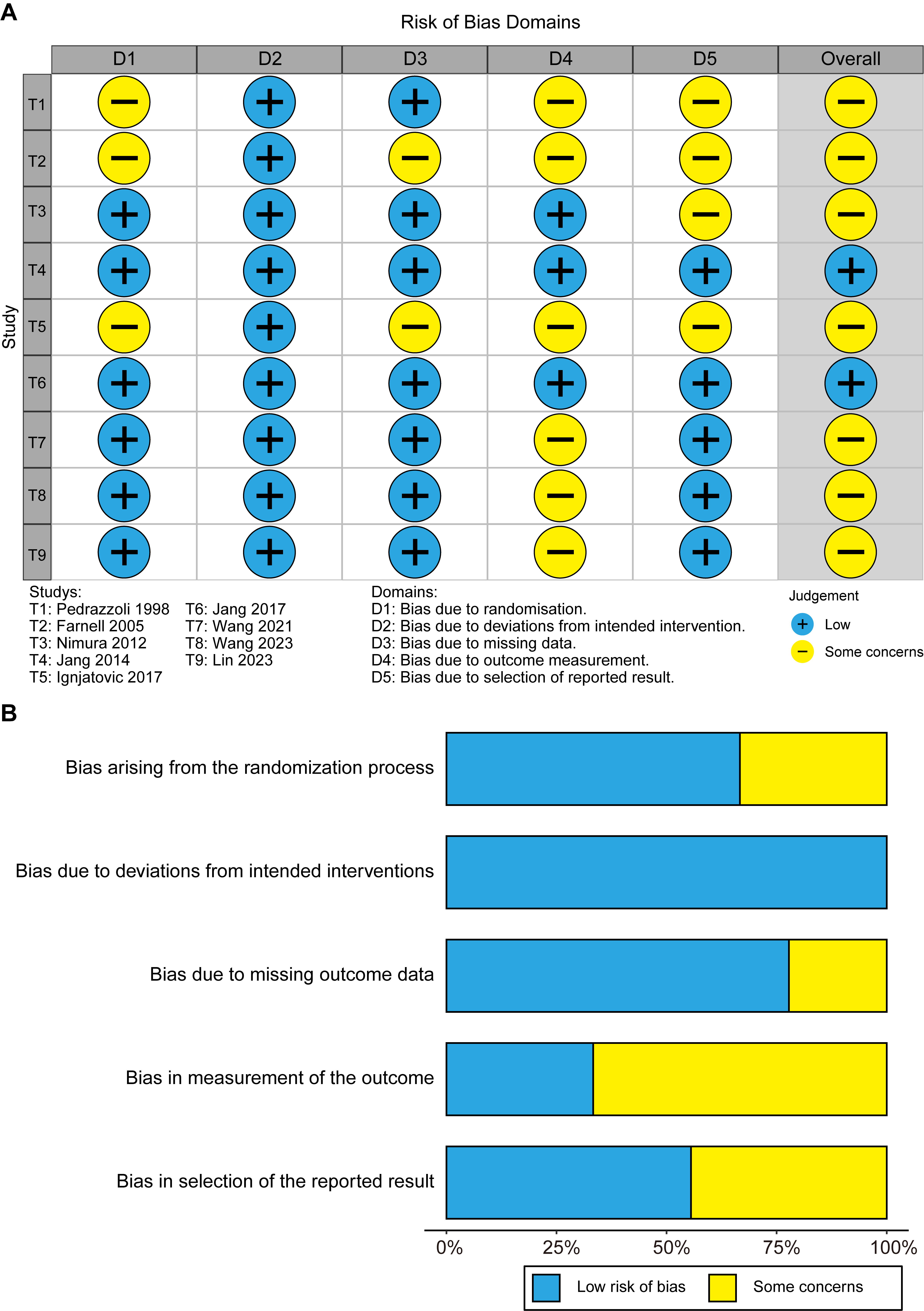
**

**Figure S1 Risk of bias for included studies.**

1. A summary table evaluating the risk of bias across five domains (D1–D5) for nine studies.
2. Bar charts illustrating the proportion of studies classified as having "low risk of bias" (blue) or "some concerns" (yellow) across each bias domain.

**
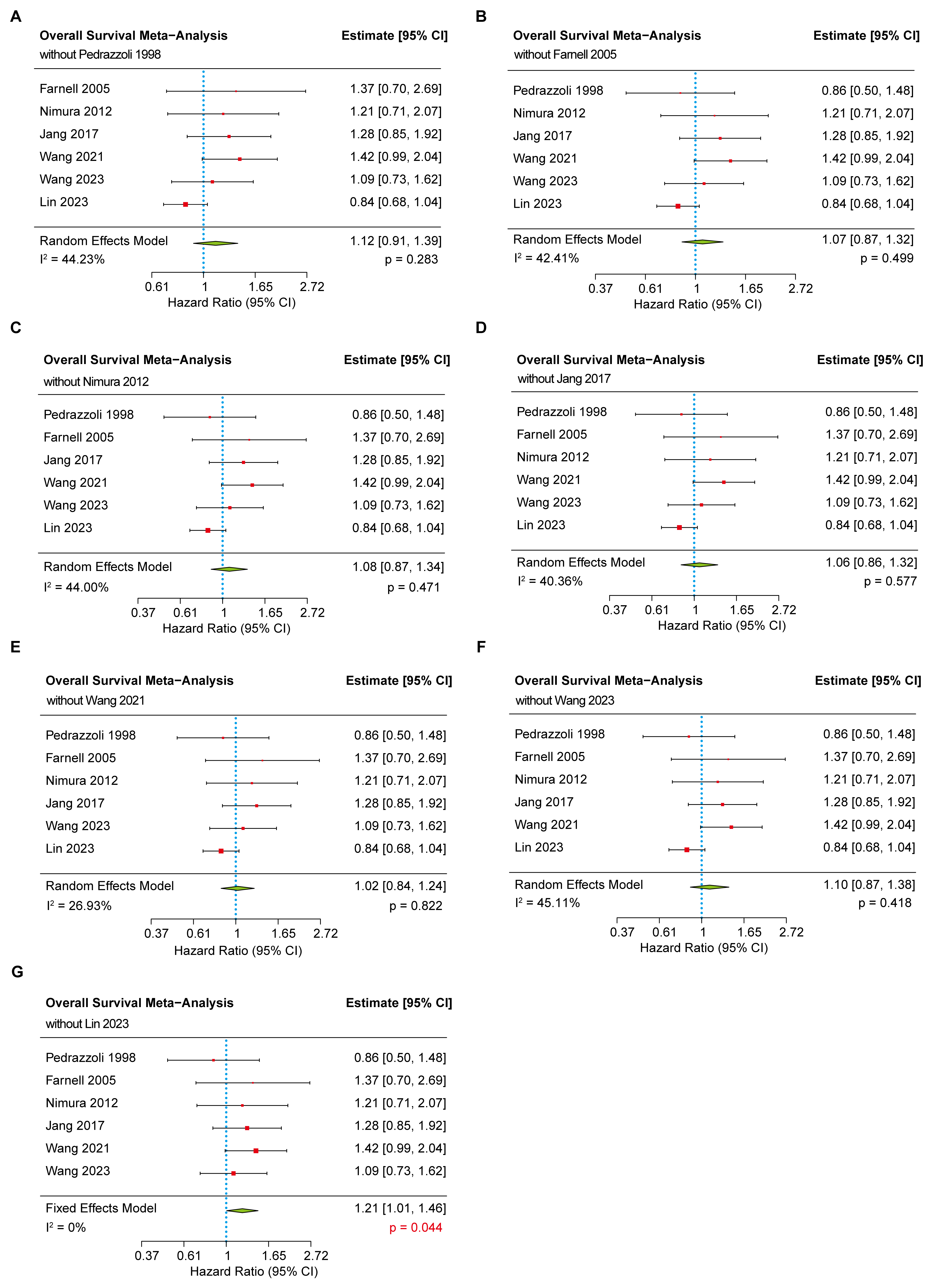
**

**Figure S2 Forests plot of the sensitive analysis of overall survival.**

1. The meta-analysis of overall survival without Pedrazzoli 1998.
2. The meta-analysis of overall survival without Farnell 2005.
3. The meta-analysis of overall survival without Nimura 2012.
4. The meta-analysis of overall survival without Jang 2017.
5. The meta-analysis of overall survival without Wang 2021.
6. The meta-analysis of overall survival without Wang 2023.
7. The meta-analysis of overall survival without Lin 2023.


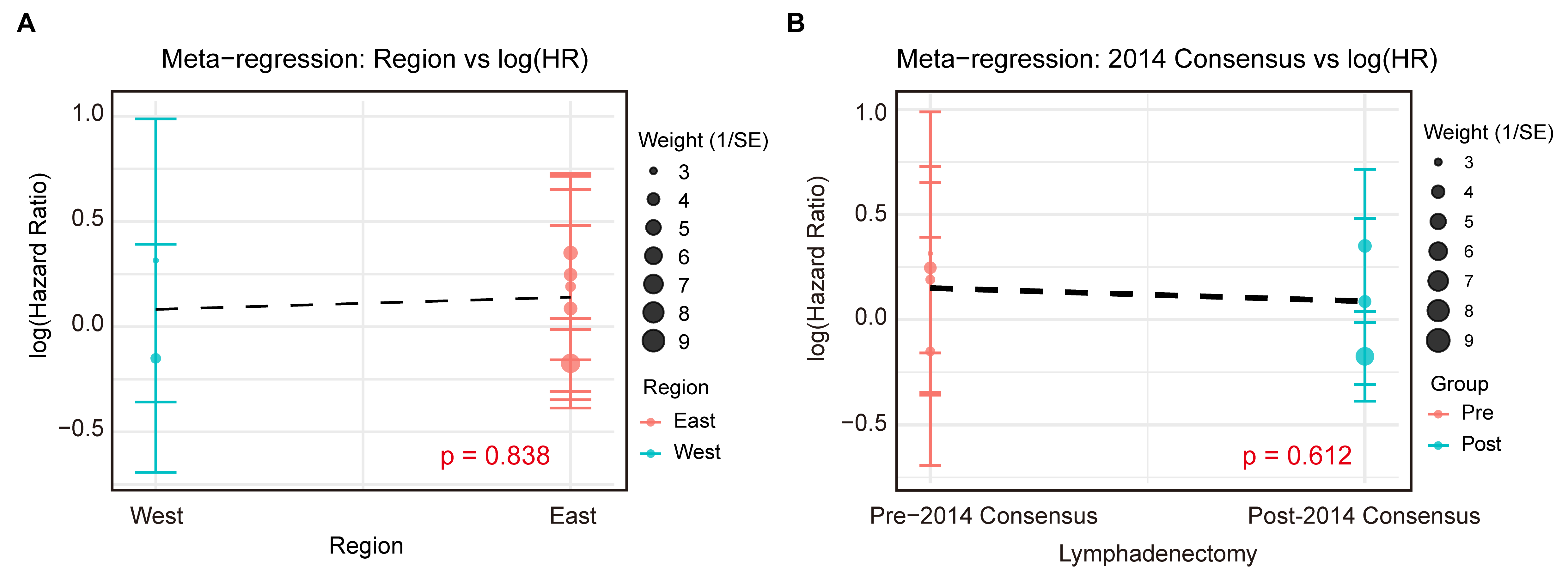


**Figure S3 Plot of meta-regression of overall survival.**

1. The meta-regression of overall survival using region as moderators.
2. The meta-regression of overall survival using adherence to the 2014 consensus guidelines for lymphadenectomy as moderators.


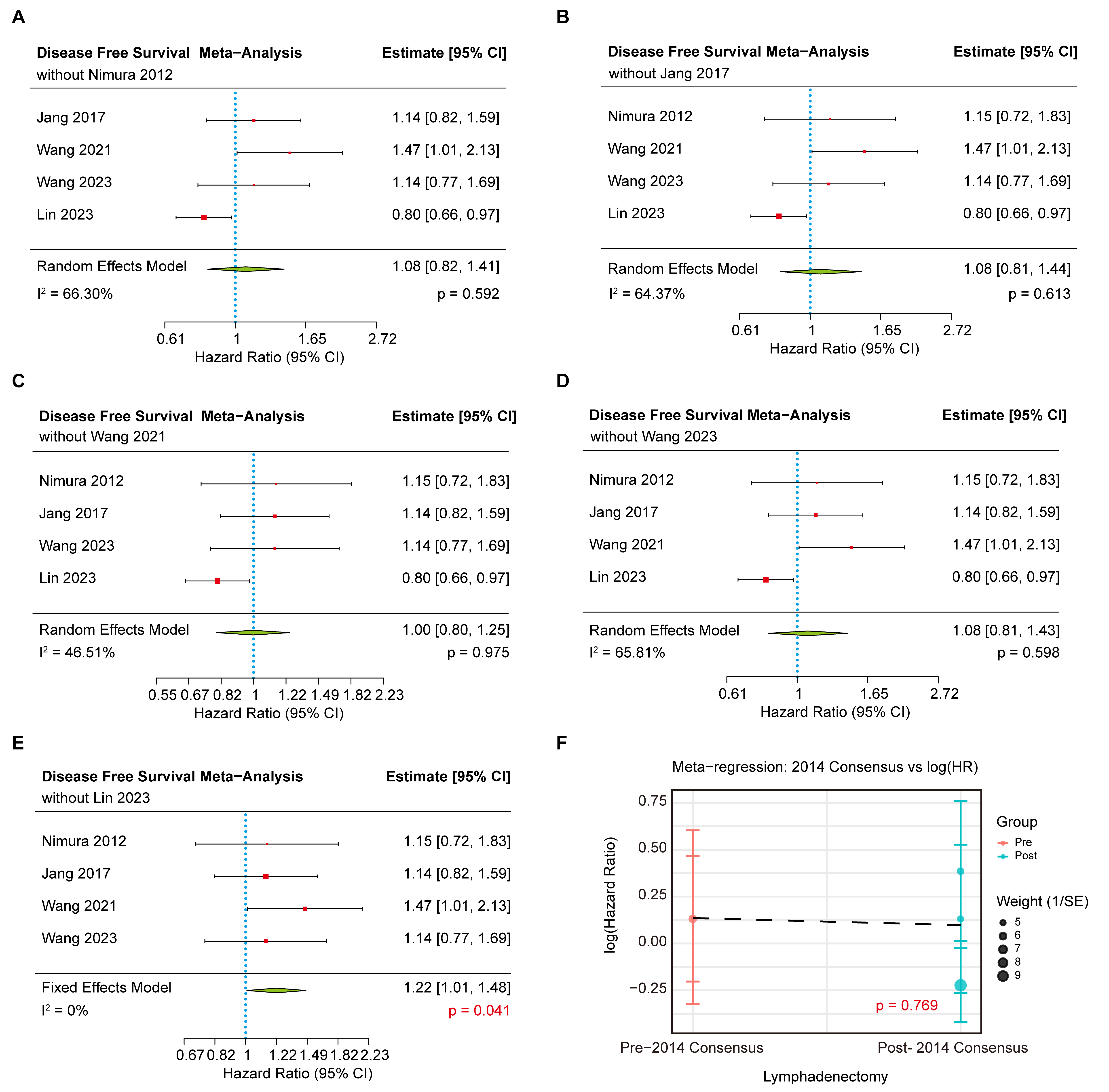


**Figure S4. Forest plots of the sensitive analysis and meta-regression of disease-free survival.**

1. The meta-analysis of overall survival without Nimura 2012.
2. The meta-analysis of overall survival without Jang 2017.
3. The meta-analysis of overall survival without Wang 2021.
4. The meta-analysis of overall survival without Wang 2023
5. The meta-analysis of overall survival without Lin 2023.
6. The meta-regression of disease-free survival using adherence to the 2014 consensus guidelines for lymphadenectomy as moderators.


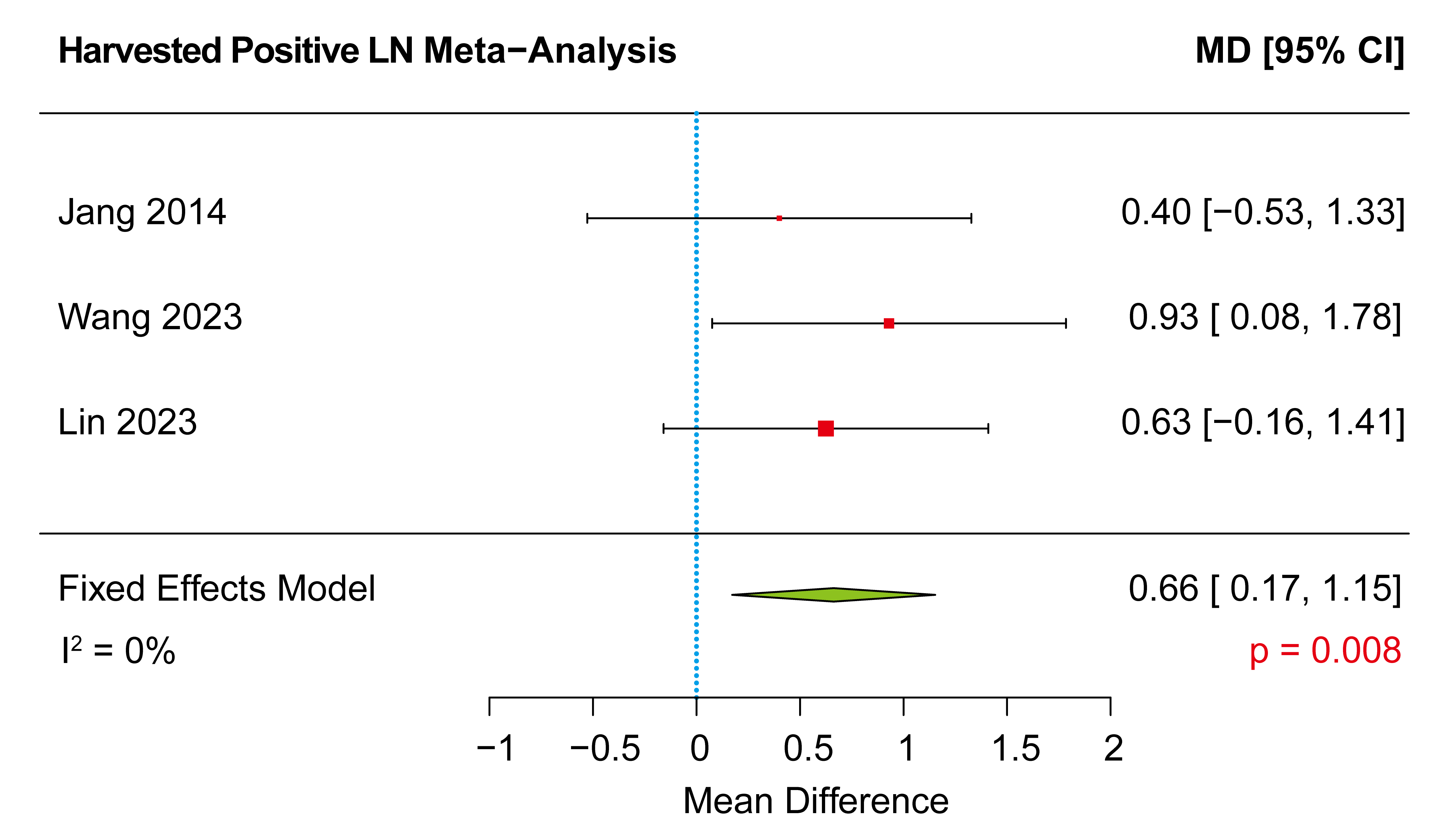


**Figure S5. The forest plot showing the meta-analysis of number of harvested positive lymph nodes between EPD and SPD group.**


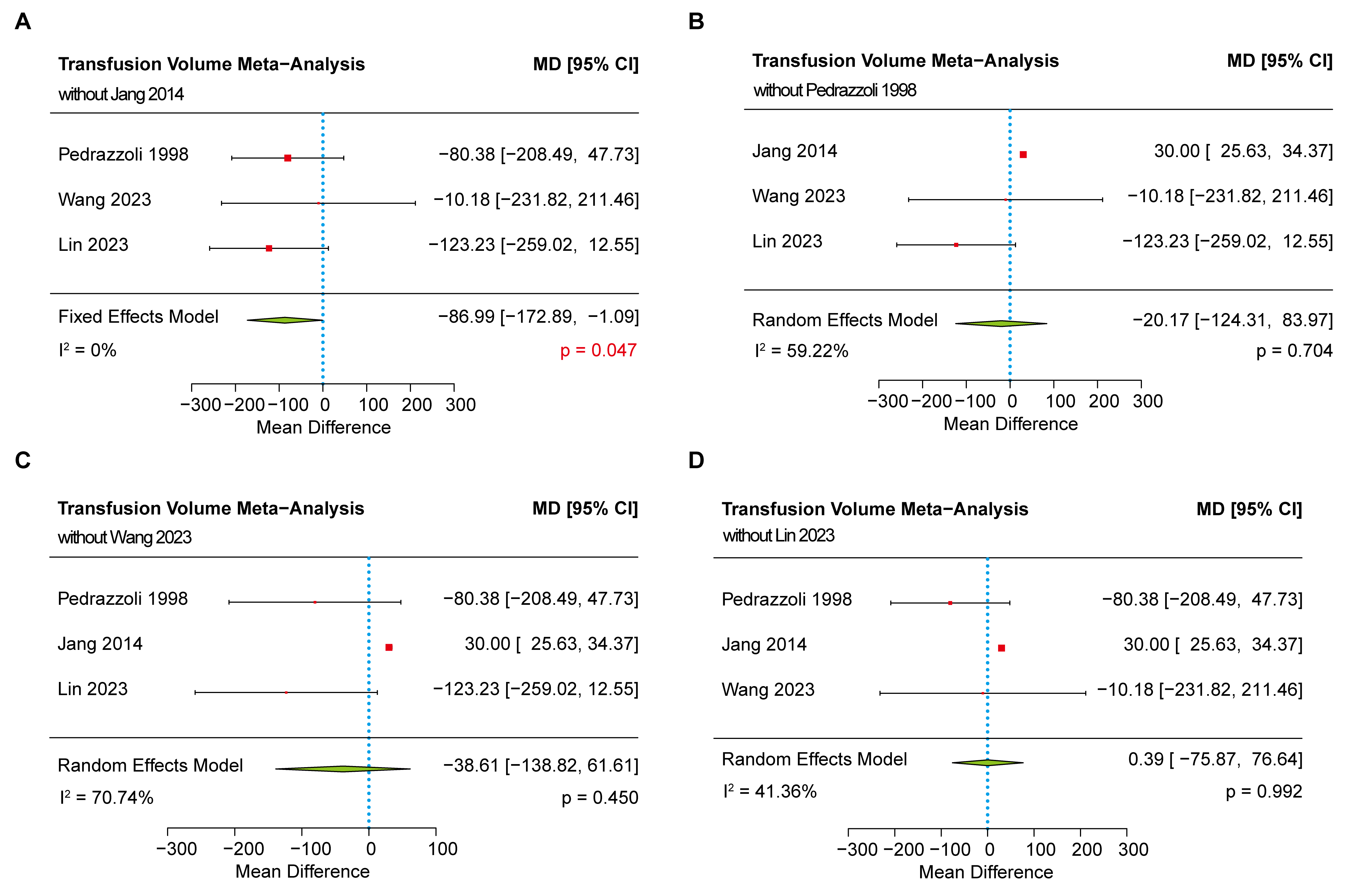


**Figure S6. Forest plots of the sensitive analysis of transfusion volume.**

1. The meta-analysis of overall survival without Jang 2014.
2. The meta-analysis of overall survival without Pedrazzoli 1998.
3. The meta-analysis of overall survival without Wang 2023.
4. The meta-analysis of overall survival without Lin 2023.

**Table S1 The extents of lymphadenectomy in the standard and extended groups in pancreatoduodenectomy**

| Lymph node station | Pedrazzoli et al 1998 | | Farnell et al 2005 | | Nimura et al 2012 | | Jang et al 2014 | | Ignjatovic et al 2017 | | Wang et al 2021 | | Wang et al 2023 | | Lin et al 2023 | |
| --- | --- | --- | --- | --- | --- | --- | --- | --- | --- | --- | --- | --- | --- | --- | --- | --- |
|  | Standard | Extended | Standard | Extended | Standard | Extended | Standard | Extended | Standard | Extended | Standard | Extended | Standard | Extended | Standard | Extended |
| 5 | YES | YES | YES | YES | NO | NO | NO | YES | NO | NO | YES | YES | YES | YES | YES | YES |
| 6 | YES | YES | YES | NO | NO | NO | NO | YES | NO | NO | YES | YES | YES | YES | YES | YES |
| 7 | —— | —— | —— | —— | —— | —— | —— | —— | —— | —— | —— | —— | —— | —— | —— | —— |
| 8a | YES | YES | YES | YES | NO | YES | NO | YES | YES | YES | YES | YES | YES | YES | YES | YES |
| 8p | YES | YES | NO | YES | NO | YES | NO | YES | NO | YES | NO | NO | NO | YES | NO | YES |
| 9 | NO | YES | NO | YES | NO | YES | NO | YES | NO | YES | NO | YES | NO | NO | NO | YES |
| 11 | —— | —— | —— | —— | —— | —— | —— | —— | —— | —— | —— | —— | —— | —— | —— | —— |
| 12a | NO | YES | NO | YES | NO | YES | YES | YES | NO | YES | NO | NO | NO | YES | NO | YES |
| 12b | YES | YES | YES | YES | NO | YES | NO | YES | YES | YES | YES | YES | YES | YES | YES | YES |
| 12c | NO | YES | YES | YES | NO | YES | NO | YES | YES | YES | YES | YES | YES | YES | YES | YES |
| 12p | NO | YES | NO | YES | NO | YES | YES | YES | NO |  | NO | YES | NO | YES | NO | YES |
| 12h | NO | NO | NO | NO | NO | NO | NO | YES | NO | NO | NO | NO | NO | NO | NO | YES |
| 13 | YES | YES | YES | YES | YES | YES | YES | YES | YES | YES | YES | YES | YES | YES | YES | YES |
| 14a | NO | YES | YES | YES | NO | YES | NO | YES | YES | YES | YES | YES | YES | YES | YES | YES |
| 14b | NO | YES | YES | YES | NO | YES | NO | YES | YES | YES | YES | YES | YES | YES | YES | YES |
| 14c | NO | YES | NO | YES | NO | YES | NO | YES | NO | YES | NO | YES | NO | YES | NO | NO |
| 14d | NO | YES | NO | YES | NO | YES | NO | YES | NO | NO | NO | YES | NO | YES | NO | NO |
| 14p | NO | YES | NO | YES | NO | YES | NO | NO | NO | NO | NO | NO | NO | NO | NO | NO |
| 16a | NO | YES | NO | NO | NO | YES | NO | YES | NO | NO | NO | YES | NO | YES | NO | YES |
| 16b | NO | YES | NO | YES | NO | NO | NO | YES | NO | NO | NO | YES | NO | YES | NO | YES |
| 17 | YES | YES | YES | YES | YES | YES | YES | YES | YES | YES | YES | YES | YES | YES | YES | YES |
| 18 | YES | YES | NO | NO | NO | NO | NO | NO | NO | NO | NO | NO | NO | NO | NO | NO |
| celiac trunk | NO | YES | NO | YES | NO | YES | NO | YES | NO | YES | NO | YES | —— | —— | NO | YES |
| SMA | NO | YES | YES | YES | NO | YES | NO | YES | NO | YES | NO | YES | —— | —— | NO | YES |

| **Author** | **Study group** | **Number of patients** | **CEA** | **CA19-9** | **Tumor size** | **T stage** | **Tumor differentiation** | **TNM stage** |
| --- | --- | --- | --- | --- | --- | --- | --- | --- |
| Pedrazzoli et al 1998 | Standard | 40 | NA | NA | 3.43 ± 1.28 | NA | 5/26/9 | 14/2/24 |
|  | Extended | 41 | NA | NA | 2.95 ± 0.81 | NA | 12/21/8 | 14/3/24 |
| Farnell et al 2005 | Standard | 40 | NA | NA | NA | NA | NA | NA |
|  | Extended | 39 | NA | NA | NA | NA | NA | NA |
| Nimura et al 2012 | Standard | 51 | NA | NA | NA | NA | NA | NA |
|  | Extended | 50 | NA | NA | NA | NA | NA | NA |
| Jang et al 2014 | Standard | 83 | 4.1 ± 5.3 | 677.9 ± 1720.7 | 2.98 ± 0.84 | 8/2/73 | NA | 7/76/0 |
|  | Extended | 86 | 3.9 ± 5.8 | 996.9 ± 3037.7 | 3.12 ± 0.91 | 3/3/80 | NA | 3/83/0 |
| Ignjatovic et al 2017 | Standard | 30 | NA | NA | NA | NA | NA | NA |
|  | Extended | 30 | NA | NA | NA | NA | NA | NA |
| Jang et al 2017 | Standard | 83 | 4.1 ± 5.3 | 677.9 ± 1720.7 | 2.98 ± 0.84 | 8/2/73 | NA | 7/76/0 |
|  | Extended | 86 | 3.9 ± 5.8 | 996.9 ± 3037.7 | 3.12 ± 0.91 | 3/3/80 | NA | 3/83/0 |
| Wang et al 2021 | Standard | 79 | 4.12 ± 3.32 | 281.16 ± 402.47 | 3.21 ± 1.13 | 6/58/15 | NA | 32/33/14 |
|  | Extended | 74 | 3.51 ± 2.40 | 360.19 ± 595.75 | 3.18 ± 1.13 | 5/57/12 | NA | 26/36/12 |
| Wang et al 2023 | Standard | 81 | 5.20 ± 6.22 | 425.54 ± 993.65 | 3.03 ± 0.90 | NA | NA | 29/36/16 |
|  | Extended | 89 | 6.19 ± 9.09 | 678.88 ± 1771.10 | 3.17 ± 0.95 | NA | NA | 24/32/33 |
| Lin et al 2023 | Standard | 199 | 47.23 ± 229.71 | 595.26 ± 2195.17 | 3.35 ± 1.68 | 7/21/171 | 8/94/97 | 13/186/0 |
|  | Extended | 201 | 17.80 ±77.14 | 724.49 ± 2917.57 | 3.08 ± 1.41 | 10/20/171 | 14/108/79 | 19/182/0 |
| NA: not available; CEA: mean ± SD, ng/ml; CA19-9: mean ± SD, U/ml; Tumor size: mean ± SD, cm; T stage: T1/T2/T3; Tumor differentiation: Well/Moderate/Poor; TNM stage: I/II/III | | | | | | | | |

**Table S2 Characteristics of tumors of included studies**
